# Mapping the implementation process and outcomes of HIV counselling and testing programmes in low -middle-income countries: a scoping review protocol

**DOI:** 10.3389/fpubh.2026.1902498

**Published:** 2026-09-23

**Authors:** Danemore Mapuranga, Margaret Ntombizodwa Mbelle, Nametso Lebogang Patience Segomotso, Mathildah Mpata Mokgatle

**Affiliations:** Department of Public Health, School of Health Care Sciences, Sefako Makgatho Health Sciences University, Ga-Rankuwa, South Africa

**Keywords:** HIV counselling and testing, HIV/AIDS, low-middle income countries, monitoring, outcomes, process evaluation, programme implementation

## Abstract

**Background:**

HIV counselling and testing (HCT) programmes remain a cornerstone of global HIV prevention, treatment, and care strategies, particularly in low- and middle-income countries (LMICs) where the burden of HIV is disproportionately high. Despite progress in expanding access to testing services, gaps persist in implementation, uptake, and effectiveness of HCT interventions. Variations in health system capacity, sociocultural factors, stigma, and resource constraints continue to influence programme delivery and experiences. Understanding implementation processes and outcomes is essential for identifying best practices and informing policy and practice. This scoping review seeks to map evidence on implementation processes and outcomes of HCT programmes in LMICs, highlighting challenges, facilitators, and areas requiring further research.

**Aim:**

To map existing evidence on the implementation processes and outcomes of HIV Counselling and Testing programmes in LMICs, identify knowledge gaps, and inform policy, practice, and research.

**Method:**

This systematic scoping review will follow the Joanna Briggs Institute methodology, the Arksey and O’Malley framework, and PRISMA. Grey literature relevant to HIV Testing Services implementation in LMICs will also be reviewed. Searches will be conducted in Embase, Global Health, African Index Medicus, PubMed, and Scopus for studies published between 2010 and 2025. Articles will be screened independently using predefined inclusion and exclusion criteria before full-text review and data extraction. Extracted data will be analysed using descriptive and thematic content analysis. Information on study characteristics, implementation strategies, target populations, barriers, facilitators, and outcomes will be charted. Findings will be synthesized narratively and presented using tables, charts, and thematic maps. Ethical approval is not required as the review uses publicly available literature.

**Findings:**

The review will provide a comprehensive overview of implementation processes and outcomes of HCT programmes in LMICs, identify evidence gaps, and inform future HIV policy, implementation strategies, and research.

**Funding:**

The review is part of a PhD project, Department of Public Health, Sefako Makgatho Health Sciences University. This research received no specific grant from funding agencies in the public, commercial, or not-for-profit sectors.

**Panel:**

Research in context.

**Evidence before this study:**

Before undertaking this scoping review, a comprehensive search of the literature will be conducted in electronic databases including Embase, Global health, African Index Medicals and PubMed. Additional sources will include reference lists of eligible studies and relevant grey literature. The search will include studies published from January 2000 to December 2025 using English Language. Search terms will include combinations of keywords and Medical Subject Headings (MeSH) related to “HIV counselling and testing”, “implementation,” “programme outcomes,” “barriers,” “facilitators,” “uptake,” “service delivery,” “low- and middle-income countries,” and related terms. Studies will be screened according to predefined inclusion and exclusion criteria focusing on HIV counselling and testing (HCT) programme implementation and outcomes in LMICs. The quality and methodological characteristics of the included studies will be described to provide context for interpreting the available evidence. As this is a scoping review, quantitative pooling or meta-analysis of findings will not be undertaken.

**Added value of this study:**

This scoping review will provide a comprehensive mapping of the implementation processes and outcomes of HIV counselling and testing programmes across low- and middle-income countries. The review will synthesize evidence on implementation strategies, programme uptake, service utilization, barriers and facilitators, quality of services, accessibility, sustainability, and scalability of HCT programmes. It will also identify gaps in the literature and variations in implementation approaches across different settings and populations. The findings are expected to generate a broader understanding of how HCT programmes are implemented and what factors influence their effectiveness in LMIC contexts.

**Implications of all the available evidence:**

The findings from this scoping review, together with the existing evidence, will help inform policy makers, programme implementers, healthcare providers, and researchers on effective approaches for strengthening HIV counselling and testing programmes in LMICs. The review will highlight implementation challenges and successful strategies that may support improvements in accessibility, quality, uptake, and sustainability of HCT services. In addition, the review will identify evidence gaps and priorities for future research aimed at enhancing HIV prevention, early diagnosis, linkage to care, and overall health system responses to HIV in resource-limited settings.

## Introduction

HIV prevention remains one of the world’s top public health and development priorities (1). HIV remains a major global public health concern, disproportionately affecting populations in Low- and Middle-Income Countries (LMICs), where access to timely diagnosis is essential for prevention, treatment initiation, and epidemic control (1). HIV Counselling and Testing (HCT), now widely delivered as HIV Testing Services (HTS), represents the critical entry point to the HIV care cascade and is central to achieving global targets such as the UNAIDS 95/95/95 goals. Over the past two decades, many LMICs have developed and implemented national HIV/AIDS Strategic Plans to guide the scale-up, integration, and sustainability of testing services within health systems. While substantial progress has been made in expanding coverage, less is known about how these strategic plans have been operationalized in practice, including the processes, implementation strategies, and system-level factors influencing programme performance. Understanding implementation processes and outcomes such as acceptability, adoption, fidelity, coverage, sustainability, and scalability is essential to identify gaps between policy and practice and to inform future strategic planning.

Globally, approximately 36^.^9 million people are living with HIV/AIDS (2). Sub-Saharan Africa carries a disproportionate burden of HIV/AIDS, accounting for more than 70% of the global burden (3). Despite the gains in HIV prevention, new HIV infections continue to be a major public health challenge in the Eastern, Central and Southern Africa region (4). In 2022, UNAIDS estimated 450,000 new HIV infections in eastern and southern Africa and 190,000 new infections in western and central Africa (4). A high proportion of undiagnosed HIV is likely to be undermining efforts to reduce HIV transmission (5).

HIV testing is crucial for controlling the epidemic because it is the primary entry point to both prevention and treatment (4). Knowing one’s HIV status enables early initiation of antiretroviral therapy, which suppresses viral load, improves individual health, and effectively prevents onward transmission (treatment as prevention). HIV counselling and testing is regarded as a prevention strategy with the assumption that knowing one’s status is effective in enabling people to make risk reducing changes in their behaviour. Eradicating the HIV epidemic by 2020 was one of the key target of the Sustainable Development Goal on health and well-being (6, 7, 12).

At a population level, widespread testing provides essential data for surveillance, guiding resource allocation and public health strategies, and is therefore a cornerstone of efforts to reduce new infections and achieve epidemic control. A scoping review is the most appropriate approach for evaluating HIV /AIDS Policies for low to medium income countries to map and synthesize a broad range of existing evidence to assess the effectiveness of HIV testing to inform policy and future research. Therefore, this scoping review aims to map existing evidence on the implementation processes and outcomes of HIV Counselling and Testing programmes in LMICs, in order to identify knowledge gaps and inform policy, practice, and research.

## Methods

We will conduct a scoping review on the implementation of HIV Testing Services in Low-Medium Income Countries in using the JBI methodology, Preferred Reporting Items for Systematic Reviews (8), and Meta-Analyses extension for Scoping Reviews (PRISMA-ScR) guidelines (9, 10). Grey literature relevant to the implementation of the HIV Testing Services in Low-Medium Countries (LMICs) will be systematically reviewed to capture policy, programmatic, and implementation evidence not available in peer-reviewed sources will be utilized. The six stages of Arksey and O’Malley will be used in undertaking this scoping review: (1) defining the research question; (2) developing the inclusion and exclusion criteria; (3) describing the search strategy; (4) searching for the evidence; (5) selecting the evidence; (6) extracting the evidence. This strategy will encompass a broad range of keywords and synonyms related to implementation of HIV Testing Services in LMICs. Additionally, we will employ the Population, Concept, and Context Framework (PCC) to formulate our eligibility criteria and direct the study selection process.

### Stage 1: defining the research question

Through consultation with the research team and key stakeholders, the overall main research question will be defined as: For the purpose of this review, implementation research has been defined within the broader frameworks of implementation research, knowledge translation and evidence informed decision-making. Based on the primary research question, the following specific research questions will be used:

1. How have HIV counselling and testing (HTS) programmes been designed, implemented, and monitored within National HIV/AIDS Strategic Plans in low- and middle-income countries?
2. What implementation strategies have been used across different HIV Testing Service modalities in low- and middle-income countries?
3. What implementation outcomes have been reported for HIV Testing Services programmes in low- and middle-income countries?
4. What facilitators and barriers influence the implementation of HIV Testing Services programmes at policy, health system, community, and provider levels in low- and middle-income countries?
5. What evidence gaps exist in the implementation of HIV Testing Services programmes across different modalities and implementation outcomes in in low- and middle-income countries?

### Stage 2: developing the inclusion and exclusion criteria

#### Inclusion criteria

This scoping review will focus on the implementation of HIV strategic plans in Low- and Middle-Income Countries as classified by the World Bank at the time of the study. These include activities such as coverage, acceptability, fidelity assessment, adoption and penetration, implementation of HIV Counselling and Testing, HIV Testing Services, or HIV Self-Testing and factors influencing acceptability and uptake of HIVST (11). Under the population element, studies that examines the implementation processes and outcomes of HIV Counselling and Testing (HCT)/HIV Testing Services (HTS) programmes, including the following service delivery models: facility-based, community-based, self-testing, index testing, key population models, integrated models, workplace, schools, and tertiary institutions will be included. Participants will include health workers, programme implementers, policy makers and populations receiving HIV Counselling Testing services/HIV Testing services.

Under the concept element, studies addressing implementation of HCT programmes, including programme fidelity and adaptations, uptake and coverage of testing services, linkage to care after testing, sustainability of programmes and integration into routine health systems.

On the context element, studies conducted in real-world healthcare, community, and outreach settings, Low- and middle-income countries (LMICs) and studies aligned with HIV/AIDS strategic plans or programmatic contexts will be included. Time Frame, publications from 2010 to 2025 will be utilised. Language studies published in English only. Quantitative studies, qualitative studies, mixed-methods studies, implementation studies, process evaluations, programme evaluations, observational studies and case studies will be utilised. Peer-reviewed journal articles and Grey literature, including: strategic plans, programme evaluation reports, policy briefs, Government and Non-Governmental Organisation reports will be utilised.

### Exclusion criteria

Excluded will be studies focusing on issues other than HIV testing services or HCT programmes. Studies that do not address implementation processes, programme delivery, or outcomes of HCT programmes. Studies conducted exclusively in high-income countries without relevance to real-world service delivery. Studies published before 2010 or after 2025 will be excluded. Publications in languages other than English will be excluded. On Study designs, editorials, commentaries, opinion pieces, letters to the editor without primary data will be excluded. On Sources/Evidence types element, sources lacking sufficient methodological detail or empirical evidence will be excluded. See Table 1 for the population concept and context framework.

**Table 1:** Population concept and context framework.

PCC Element	Inclusion criteria	Exclusion criteria
Population (P)	-Implementation processes and outcomes of HCT programmes including: -Service delivery models (facility-based, community-based, self-testing, index testing, Key population models, Integrated models, workplace, schools, and tertiary institutions). -Health workers, programme implementers, policy makers, and populations receiving HCT services	-Studies focusing on other issues rather than HIV testing services/HIV Counselling Testing programmes.
Concept (C)	Implementation of HCT programmes including: -Programme fidelity and adaptations -Uptake and coverage of testing services -Linkage to care after testing -Sustainability of programmes -Integration into routine health systems	-Studies not addressing implementation processes, programme delivery and outcomes
Context (C)	-HIV/AIDS strategic plans; studies conducted in real-world healthcare, community, or outreach settings in LMICs.	-Studies conducted exclusively in high-income countries not linked to real service delivery
Time window	−2010–2025 (to capture scale-up of index testing, self-testing, and community-based HIV testing modalities)	-Published outside 2010–2025 period.
Languages	-English language only	-Publications in any other language than English.
Study designs	-Quantitative, qualitative, mixed-methods, implementation studies, process evaluations, program evaluations, observational studies, case studies.	-Editorials, commentaries, opinion pieces, letters to editors without primary data.
Sources/evidence types	Peer-reviewed journals, grey literature (Strategic plan documents, Programme evaluation reports, policy briefs, government or NGO documents and strategic plan reports).	Sources lacking sufficient methodological detail/empirical evidence/systematic reviews, e.g., scoping review studies should be excluded.

HCT, HIV Counselling and Testing; HTS, HIV Testing Services. LMICs, low- and middle-income countries. NA, not applicable.

### Stage 3: describing the search strategy

The search strategy will be developed with the guidance of a reference librarian and adapted for other databases using appropriate controlled vocabulary and syntax. The search strategy incorporated a combination of Medical Subject Headings (MeSH), free-text keywords, and Boolean operators (AND, OR) to maximise coverage. The search strategy will use search terms that are sensitive enough to capture literature sources relevant to implementation science, with due cognisance of the field’s diverse and overlapping nomenclature and search. The primary information sources for this scoping review will be scientific databases, namely Embase, Global health, African Index Medicals and PubMed. The search will be conducted in April 2026. The search string, will be developed iteratively by two (2) experts, will be equally applied across the abovementioned databases. Table 2 presents the search terms that will be used across different databases for this review.

**Table 2:** Expanded and detailed search strategy.

Database	Search Strategy	Hits
Embase	(‘hiv counseling’ OR ‘hiv counselling’ OR ‘hiv testing’ OR ‘voluntary counseling and testing’ OR ‘VCT’ OR ‘HIV testing services’ OR ‘HIV counselling and testing’ OR ‘HTC’) AND (‘program implementation’ OR implementation OR ‘implementation process’ OR ‘program evaluation’ OR ‘programme evaluation’ OR outcome* OR effectiveness OR impact) AND (‘HIV strategic plan’ OR ‘national HIV strategic plan’ OR ‘HIV/AIDS strategic plan’ OR ‘HIV policy’ OR ‘HIV/AIDS policy’ OR ‘national HIV program*’) AND (‘low income countr*’ OR ‘middle income countr*’ OR ‘developing countr*’ OR ‘resource limited setting*’ OR ‘low and middle income countr*’ OR LMIC* OR Africa OR Asia OR ‘sub-Saharan Africa’)	
Global Health	(“HIV infection” OR HIV OR “human immunodeficiency virus”) AND (“HIV testing” OR “HIV counselling” OR “HIV counselling and testing” OR “voluntary counselling and testing” OR VCT OR HTC) AND (“program implementation” OR “implementation process” OR “program evaluation” OR “programme evaluation” OR outcome* OR effectiveness OR impact) AND (“HIV strategic plan” OR “national HIV program*” OR “HIV/AIDS strategic plan” OR “HIV policy” OR “HIV/AIDS policy”) AND (“developing country” OR “low income countr*” OR “middle income countr*” OR “low- and middle-income countr*” OR LMIC* OR “resource-limited setting*” OR “sub-Saharan Africa” OR Asia)	
African Index Medicus	(“HIV infection” OR HIV OR “human immunodeficiency virus”) AND (“HIV testing” OR “HIV counselling” OR “HIV counselling and testing” OR “voluntary counselling and testing” OR VCT OR HTC) AND (“program implementation” OR “implementation process” OR “program evaluation” OR “programme evaluation” OR outcome* OR effectiveness OR impact) AND (“HIV strategic plan” OR “national HIV program*” OR “HIV/AIDS strategic plan” OR “HIV policy” OR “HIV/AIDS policy”) AND (“low income countr*” OR “middle income countr*” OR “low- and middle-income countr*” OR LMIC* OR “resource-limited setting*” OR Africa OR “sub-Saharan Africa”).	
Pub Med	(“HIV Infections”[Mesh] OR “Acquired Immunodeficiency Syndrome”[Mesh] OR HIV[tiab] OR AIDS[tiab] OR “human immunodeficiency virus”[tiab]) AND (“HIV Testing”[Mesh] OR “Mass Screening”[Mesh] OR”Counseling”[Mesh] OR “hiv counselling and testing”[tiab] OR “HIV testing”[tiab] OR “voluntary counselling and testing”[tiab] OR “HIV self-testing”[tiab] OR “self-testing”[tiab] OR “index testing”[tiab] OR “community-based testing”[tiab] OR” facility-based testing”[tiab])AND(“Implementation Science”[tiab] OR “Program Evaluation”[Mesh] OR “Process Assessment (Health Care)”[Mesh] OR implementation[tiab]OR “program implementation”[tiab] OR “programme implementation”[tiab] OR fidelity[tiab] OR adaptation*[tiab] OR uptake[tiab] OR coverage[tiab] OR “linkage to care”[tiab] OR sustainability[tiab] OR integration[tiab]) AND (“Health Policy”[Mesh] OR “Strategic Planning”[Mesh] OR “National Health Programs”[Mesh] OR “HIV strategic plan”[tiab] OR “national strategic plan”[tiab] OR “health strategic plan”[tiab] OR “national HIV program”[tiab] OR “HIV response”[tiab]) AND (“Developing Countries”[Mesh] OR “low income countr*”[tiab] OR “middle income countr*”[tiab] OR “low and middle income countr*”[tiab] OR LMIC*[tiab] OR “developing countr*”[tiab] OR “sub-Saharan Africa”[tiab])	
Scopus	TITLE-ABS-KEY (“human immunodeficiency virus” OR HIV OR AIDS OR “acquired immuno deficiency syndrome”)AND (“HIV testing” OR “HIV counselling and testing” OR HCT OR “voluntary counselling and testing” OR VCT OR “HIV self-testing” OR “self-testing” OR”index testing” OR “community-based testing” OR”facility-based testing” OR “HIV screening”) AND (implementation OR “program implementation” OR “programme implementation” OR “implementation research” OR “implementation science” OR “process evaluation” OR “program evaluation” OR fidelity OR adaptation* OR uptake OR coverage OR “linkage to care” OR sustainability OR integration) AND (“strategic plan*” OR “national strategic plan*” OR “HIV strategic plan*” OR policy OR policies OR “health program*” OR “health programme*” OR “national HIV program*” OR” HIV response”) AND (“low income countr*” OR “middle income countr*” OR “low and middle income countr*” OR LMIC* OR”developing countr*” OR”sub-Saharan Africa”)	

VCT, voluntary counselling and testing. HTC, HIV testing and counselling. MeSH, Medical Subject Headings. LMIC, low- and middle-income country. NA, not applicable.

### Stage 4: searching the evidence

A comprehensive literature search will be conducted using five electronic bibliographic databases namely: Embase, Global Health, Africa Index Medicus, PubMed and Scopus. Each database will be searched from 2010 up to 2025. Eligible documents included policy and strategy documents, programme reports, monitoring and evaluation outputs, donor assessments, and unpublished academic works. Retrieved documents will be screened against predefined inclusion and exclusion criteria, managed using reference software, and assessed for quality based on authority, accuracy, relevance, timeliness, and potential bias. Findings from grey literature will be triangulated with routine health information system data and other evidence sources to enhance validity and reliability of the evaluation findings. Handsearching, citation chasing, and contacting programme implementers will also be utilised.

### Stage 5: selecting the evidence

In Stage 5, the selection of evidence will be conducted through a rigorous and transparent screening process consistent with JBI guidance and PRISMA-ScR recommendations. Full-text articles will be reviewed for eligibility. All records identified through database and grey literature searches will be imported into endnote and exported into Rayyan to initiate the screening process. Rayyan will support deduplication, screening, data extraction and quality assessment. Two independent reviewers will conduct a two-stage screening process: first, titles and abstracts will be screened against the predefined inclusion and exclusion criteria, followed by full-text screening of potentially eligible articles. As a pilot, the author will independently screen fifteen randomly selected references to ensure consistent application of the eligibility criteria. Discrepancies between reviewers will be resolved through discussion, and where necessary, consultation with a third reviewer. Studies will be included if they examine the implementation processes and/or implementation outcomes of HIV Counselling and Testing (HCT) or HIV Testing Services (HTS) programmes in Low- and Middle-Income Countries (LMICs). Reasons for exclusion at the full-text stage will be documented, and the overall study selection process will be presented using a PRISMA-ScR flow diagram to ensure transparency and reproducibility. The protocol will be registered on the Prospero.

### Stage 6: extracting the evidence

In Stage 6, data extraction (charting of the evidence) will be conducted using a standardized data-charting form developed in accordance with JBI guidance for scoping reviews. Two reviewers will independently extract relevant information from each included study, including bibliographic details (author, year, country), study design, setting, population characteristics, description of the HIV Counselling and Testing (HCT) or HIV Testing Services (HTS) programme, alignment with national HIV/AIDS strategic plans, implementation processes (e.g., rollout strategies, integration approaches, stakeholder engagement), and reported implementation outcomes (e.g., acceptability, adoption, feasibility, fidelity, coverage, sustainability, scalability, and cost-related outcomes). Any discrepancies in extracted data will be resolved through discussion or consultation with a third reviewer. The extracted data in a single Microsoft Excel spreadsheet will be organized to facilitate descriptive numerical summaries and thematic analysis, enabling systematic mapping of implementation processes and outcomes across LMIC contexts.

### Data analysis and presentation

We conducted a descriptive analysis by categorizing studies according to their goals, evaluation designs, specific programme evaluations, type of evaluation, and evaluation results. Data from the included studies will be analyzed using a combination of quantitative descriptive analysis and qualitative thematic analysis to comprehensively map the implementation processes and outcomes of HIV counselling and testing programmes in low- and middle-income countries. Quantitative analysis will summarize study characteristics such as objectives, research design, geographical setting, types of HIV counselling and testing programmes evaluated, evaluation approaches, and reported outcomes, using frequencies, proportions, and trends to identify patterns in programme implementation and evaluation. Qualitative thematic analysis will be used to identify and synthesize key themes related to programme implementation and outcomes, with extracted data coded and organized into themes such as access to HIV testing services, quality of counselling and testing, alignment with national HIV/AIDS strategic plans, implementation barriers and facilitators, and programme outcomes. Integrating both quantitative and qualitative findings will help identify common factors influencing the implementation and effectiveness of HIV counselling and testing programmes. The results will be organized according to the five main research questions guiding the review and reported in accordance with the PRISMA-ScR (Preferred Reporting Items for Systematic Reviews and Meta-Analyses Extension for Scoping Reviews) guidelines (11), with findings presented through tables, charts, and narrative summaries.

Figure 1 will present the study selection process for the scoping review using a PRISMA flow diagram in an editable format such as Microsoft Word, accounting for all records identified, screened, assessed for eligibility, and excluded with reasons for exclusion at each stage. Data extraction will include variables such as author and year of publication, country, study setting, study design, sample size, participants’ age and sex, HIV testing and counselling intervention characteristics, implementation strategies, programme outcomes, and any reported contextual factors and summary measures used across studies. Variability within and between studies will be assessed narratively. Qualitative findings will be synthesised thematically to map implementation processes and outcomes across low- and middle-income countries.

**Figure 1:**
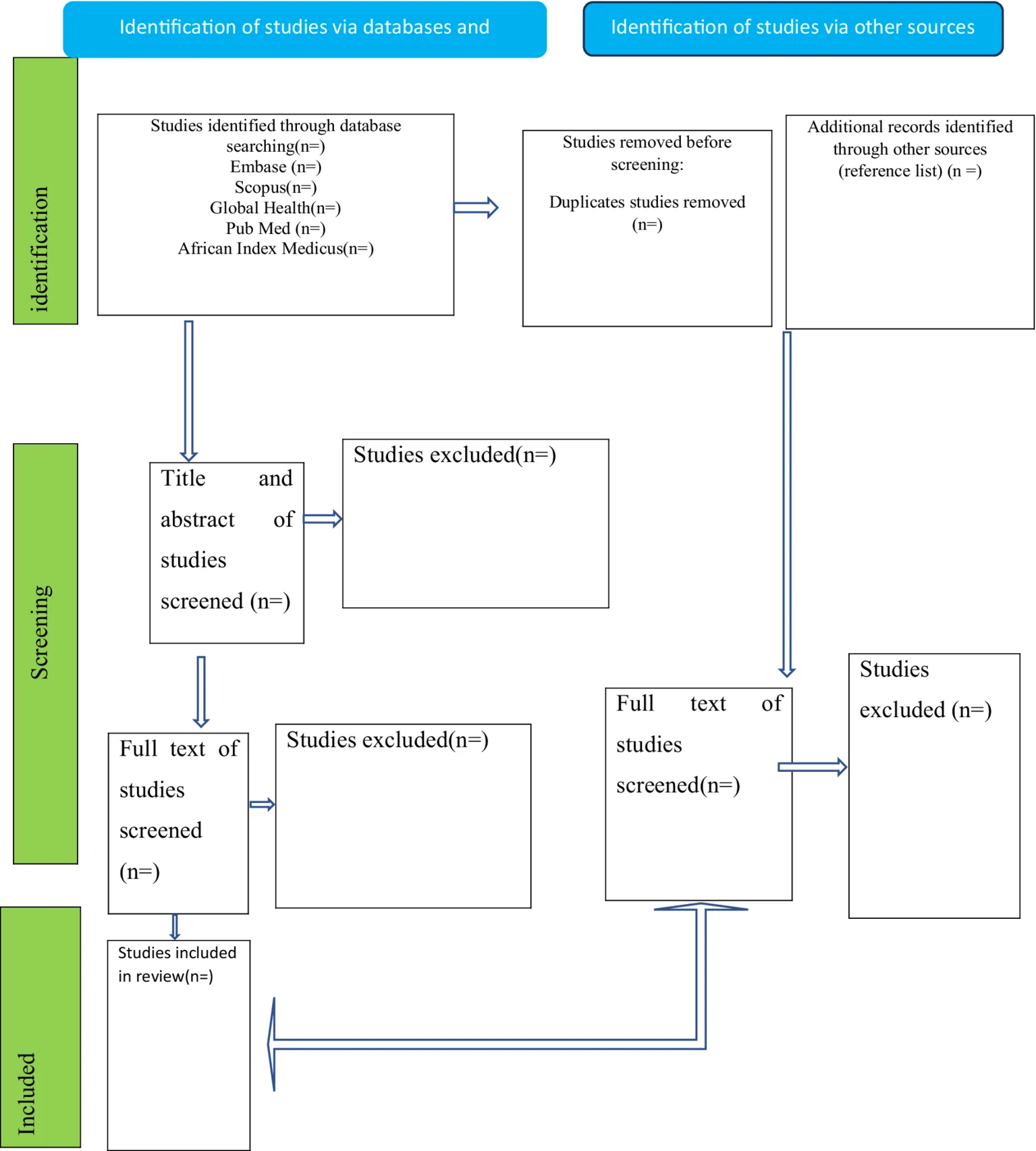
Prisma flow diagram for the scoping review process.

### Role of funding source

There was no funding source for this study. Consequently, no funder had any role in the study design, data collection, data analysis, data interpretation, or writing of the report.

## Discussion

This scoping review will map and synthesize existing evidence on the implementation processes and outcomes of HIV Counselling and Testing (HCT)/HIV Testing services (HTS) programmes in low- and middle-income countries. It will identify how programmes are designed, delivered, and evaluated across diverse contexts, highlighting variations in strategies such as community-based testing, facility-based services, mobile outreach, and self-testing approaches. The review will also examine how sociocultural, economic, and health system factors will influence programme uptake, accessibility, and sustainability.

The findings will provide an overview of reported outcomes, including testing uptake, linkage to care, early diagnosis rates, and behavioral changes following counselling. In addition, the review will explore implementation facilitators and barriers, such as stigma, workforce capacity, policy environments, funding constraints, and integration with other health services. Particular attention will be given to how programmes will address equity, especially among key and vulnerable populations.

This review will also identify gaps in the literature, including under-researched geographic regions, limited reporting on long-term outcomes, and inconsistencies in monitoring and evaluation frameworks. Through systematically mapping the available evidence, the study will contribute to a clearer understanding of what implementation approaches will be most effective and contextually appropriate in low- and middle-income countries.

### Recommendations

Based on the anticipated findings, it will be recommended that future HCT/HTS programmes will adopt context-specific and culturally sensitive implementation strategies that respond to the needs of local populations. Policymakers and programme planners will be encouraged to strengthen community engagement and reduce stigma through targeted awareness campaigns and inclusive service delivery models.

Health systems will be advised to integrate HCT/HTS services with other primary healthcare interventions to improve efficiency and continuity of care. Strengthening workforce training and support mechanisms will be essential to ensure high-quality counselling and testing services. In addition, investment in monitoring and evaluation systems will be recommended to improve data quality, standardization, and reporting of outcomes.

The review will also suggest that future research will focus on longitudinal outcomes, cost-effectiveness analyses, and the scalability of innovative testing approaches such as HIV self-testing. Greater emphasis will be placed on including marginalized and high-risk populations in both programme implementation and research efforts.

Finally, international organizations and funding bodies will be encouraged to support sustainable financing models and foster cross-country knowledge sharing to enhance the effectiveness and reach of HCT/HTS programmes in low- and middle-income countries.

### Limitations of the study

This scoping review has several limitations that should be acknowledged. First, the review will include only studies published in English Language. While this approach is practical and reflects language proficiency of the review team, it may exclude relevant evidence published in other languages from the low-and middle-income countries.

Second, the review is restricted to studies published within the predefined review period. Although this time frame was selected to capture contemporary evidence that reflects current HIV counselling and testing policies, technologies and implementation strategies, it may exclude earlier studies that made important contributions to the development of HCT implementation approaches.

Third, substantial heterogeneity is anticipated across the included studies with respect to study design, healthcare settings, target populations, implementation strategies and outcomes measures. Such diversity may limit direct comparisons between studies and preclude quantitative synthesis of the findings. Nevertheless, this variation is expected to provide a broad understanding of how HIV counselling and testing programmes have been implemented across diverse LMIC contexts.

Finally, the review will depend on the completeness and quality of reporting in the primary studies. Some implementation processes or outcomes may be insufficiently described, limiting the ability to comprehensively map all aspects of programme implementation.

### Knowledge gap and contribution of the review

Previous evidence syntheses have examined various aspects of HIV testing, including the effectiveness of HIV testing strategies, HIV self-testing, provider-initiated testing and counselling, community-based HIV testing, and linkage to HIV care. However, these reviews have generally focused on intervention effectiveness, testing uptake, or specific service delivery models rather than comprehensively examining implementation processes across low- and middle-income countries using an implementation science perspective.

Although a few scoping reviews have explored components of HIV testing programmes, they have typically been limited to specific populations (such as adolescents, pregnant women, key populations, or men), individual HIV testing approaches, and particular geographical regions. Moreover, many earlier reviews were conducted before recent developments in HIV service delivery, including differentiated service delivery models, HIV self-testing scale-up, digital innovations, community-led testing initiatives, and the updated World Health Organization recommendations. Consequently, they do not provide a comprehensive synthesis of how HIV counselling and testing programmes have been implemented across diverse LMIC settings or how implementation outcomes such as acceptability, adoption, appropriateness, feasibility, fidelity, implementation cost, penetration, and sustainability have been reported.

This scoping review seeks to address these limitations by systematically mapping the implementation processes and implementation outcomes of HIV counselling and testing programmes across LMICs. By synthesizing evidence from multiple settings and identifying implementation facilitators, barriers, and research gaps, the review will provide a comprehensive overview of current implementation practices and inform policymakers, programme managers, implementers, and researchers seeking to strengthen HIV counselling and testing services in resource-constrained settings.

## Conclusion

Findings will be shared through publication in peer-reviewed journals, policy briefs to Zimbabwe’s Ministry of Health and Child Care and National AIDS Council and presentations at public health and HIV/AIDS conferences.
